# Naringenin ameliorates myocardial injury in STZ-induced diabetic mice by reducing oxidative stress, inflammation and apoptosis *via* regulating the Nrf2 and NF-κB signaling pathways

**DOI:** 10.3389/fcvm.2022.946766

**Published:** 2022-08-10

**Authors:** Yongpeng He, Shuaiqi Wang, Hao Sun, Yan Li, Jian Feng

**Affiliations:** ^1^Chongqing Key Laboratory of Translational Research for Cancer Metastasis and Individualized Treatment, Chongqing University Cancer Hospital, Chongqing Cancer Institute, Chongqing, China; ^2^Department of Cardiology, The Affiliated Hospital of Southwest Medical University, Luzhou, China

**Keywords:** naringenin, inflammation, oxidative stress, diabetic cardiomyopathy, Nrf2, NF-κB

## Abstract

Diabetes-induced myocardial damage leads to diabetic cardiomyopathy and is closely associated with the generation of oxidative stress and inflammation. Naringenin (NG) exhibits antioxidant and anti-inflammatory effects. However, whether NG has cardioprotective effects against diabetic cardiomyopathy by regulating oxidative stress and inflammation remains unknown. This study investigated the effect of NG on diabetic cardiomyopathy based on an analysis of streptozotocin (STZ)-induced type 1 diabetic mice. The results indicated that NG reduced cardiac fibrosis and cardiomyocyte apoptosis in this diabetic model, accompanied by reduced blood glucose. NG inhibited pro-inflammatory cytokines, the level of reactive oxygen species and the expression of nuclear factor kappa-B (NF-κB), whereas the expression of antioxidant enzymes and nuclear factor erythroid 2-related factor 2 (Nrf2) were greatly enhanced by NG. Furthermore, in high glucose-treated H9C2 myocardial cells, NG effectively reduced cell apoptosis by inhibiting the formation of reactive oxygen species and pro-inflammatory cytokines. NG's antioxidant and anti-inflammatory activities were mechanistically associated with NF-κB inhibition and Nrf2 activation in animal and cell experiments. Data analysis showed that NG could regulate Nrf2 and NF-κB pathways to protect against diabetes-induced myocardial damage by reducing oxidative stress and inhibiting inflammation.

## Introduction

It is estimated that by 2040, there will be 642 million diabetes patients aged 20 to 79 years globally (1). Studies have indicated that diabetic cardiovascular complications have become the leading cause of mortality among diabetic patients (2). Some patients have a hyperglycemia-induced myocardial injury but do not have high blood pressure or coronary artery disease. This disease, termed diabetic cardiomyopathy (3), is currently of considerable interest among researchers (4). The underlying mechanisms of hyperglycemia-induced myocardial injury include cardiac inflammation, increased reactive oxygen species (ROS), interstitial fibrosis, and cardiac cell apoptosis (2, 5). Such adverse reactions induce structural remodeling and affect heart diastolic function (6). Therefore, pharmacological inhibition of inflammation, ROS, and cell apoptosis has been proven to confer cardioprotection.

Many natural flavonoids have been shown to exhibit potent cardioprotective properties (7, 8). Several lines of evidence have demonstrated that naringenin (NG), a flavonoid abundant in grapefruit, plays a valuable role in the cardiovascular system. NG has been shown to reduce cardiac damage following ischemia-reperfusion injury by inhibiting mitochondrial oxidative stress damage (9). In the mouse sepsis model, NG protected against septic cardiac dysfunction by inhibiting NF-κB-dependent cardiac inflammation (10). Furthermore, NG treatment alleviated ischemia-reperfusion injury-induced myocardial cell death in rat models (11). These studies suggest that anti-inflammatory and antioxidant properties may be involved in the cardioprotective mechanism of NG. However, it remains unknown whether NG improves diabetes-induced myocardial injury.

Therefore, this study investigated the influence of NG on myocardial injury and related signaling pathways in STZ-induced diabetic mice and high glucose-induced H9C2 cells.

## Materials and methods

### Animals and treatment

Animal experiments followed the protocol previously described with minor changes (12, 13). In this study, C57BL/6 mice (Chengdu Experimental Animals Co., Ltd., Chengdu, China) were kept at room temperature of 23 ± 1°C with a 12:12 h light: dark cycle. For the experimental protocol, the *in vivo* experiment was conducted based on that stated by the National Institutes of Health for the care and use of laboratory animals (NIH Publications No. 8023, revised 1978).

After 1 week of adaptive feeding, the animals were randomly assigned to five groups (six mice per group): control group (Ctrl), diabetes mellitus group (DM), 25 mg/kg NG + DM group (LNG + DM), 50 mg/kg NG + DM group (MNG+DM), and 75 mg/kg NG + DM group (HNG + DM). Following 12 h of fasting, 80 mg/kg of 1% STZ (Sigma, St. Louis, MO, USA) was injected intraperitoneally in diabetic mice groups (DM with/without treatment). The mice of the Ctrl group received an intraperitoneal injection with an equal volume of sodium citrate buffer. After 72 hours, the tail blood was sampled. The criterion for successfully establishing the model was a blood glucose level >16.7 mmol/L. Following the establishment of the model, different doses of NG (25, 50, and 75 mg/kg; Civi Chemical Technology Co., Ltd., Shanghai, China) were administered by gavage to the respective NG groups once daily. After euthanasia of the mice, the hearts were fixed in 4% paraformaldehyde, prepared in paraffin, and sections of 4 μm thick were cut. Fresh heart tissues were used for Western blot detection after storage at −80°C.

### Analysis of myocardial histology

Hematoxylin and eosin (HE) and Masson staining was performed and images of the stained paraffin sections were obtained using a light microscope.

### Determination of malondialdehyde (MDA) and superoxide dismutase (SOD) levels

The total protein concentration was determined using the bicinchoninic acid assay (BCA) method. The MDA and SOD levels in the cardiac tissues were detected using test kits from the Jiancheng Bioengineering Institute (Nanjing, China).

### Analysis of immunohistochemistry

The paraffin sections were deparaffinized and placed in citrate buffer. The sections were heated to boiling when the power was switched off. After 5 min, the process was repeated. After cooling, the sections were washed twice with phosphate-buffered saline (PBS) for 5 min to retrieve antigens. The sections were blocked with goat serum for 20 min at room temperature and incubated with interleukin (IL)-6 (1:100), tumor necrosis factor (TNF)-α (1:100), or NF-κB p65 (1:100) antibodies at 4°C overnight. A secondary antibody was applied for 30 min at 37°C. The sections were washed three times with PBS for 5 min, and diaminobenzidine (DAB) was used to develop the color. Finally, sections were observed, and images were obtained using a light microscope (OLYMPUS BX53, Japan). For NF-κB p65 and Nrf2 fluorescence staining, fixed cardiomyocytes were incubated with NF-κB p65 (1:100) or Nrf2 (1:100) antibodies overnight at 4°C followed by phycoerythrin (PE)-conjugated secondary antibody (1:200). The analysis of fluorescent images was performed using a fluorescence microscope.

Apoptosis in tissue or cells was quantified using the TUNEL apoptosis test kit from Beyotime Biotechnology (Jiangsu, China) according to the manufacturer's instructions. Fluorescence microscopy was performed to analyze the samples.

### Western blot

The tissues and cells were washed twice using ice-cold PBS and lysed in lysis buffer. Lysates were separated by sodium dodecyl sulfate-polyacrylamide (10%−12%) gel electrophoresis and transferred to polyvinylidene fluoride membranes. The membranes were washed with Tris-buffered saline/Tween-20 (TBST) and blocked in 5% skim milk powder dissolved in TBST for three hours, followed by the incubation with the respective primary antibody at dilutions according to the supplier's instructions.

The membranes were examined using an anti-NF-κB p65 antibody (1:1,000, Cell Signaling Technology), anti-heme oxygenase-1 (HO-1) antibody (1:1,000, Abcam), anti-Nrf2 antibody (1:1,000, Cell Signaling Technology), anti-Bax antibody (1:500, Santa Cruz Biotechnology), anti-cleaved caspase-3 antibody (1:500, Santa Cruz Biotechnology), anti-IL-6 antibody (1:500, Santa Cruz Biotechnology), anti-TNFα antibody (1:500, Santa Cruz Biotechnology), anti-nicotinamide adenine dinucleotide phosphate oxidase 2 (NOX2) antibody (1:500, Santa Cruz Biotechnology), or anti-NQO1 antibody (1:500, Abcam). Following conjugation to horseradish peroxidase, the corresponding immunoglobulin G secondary antibody (1:1,000, Beyotime Biotechnology) was used to detect the primary antibodies. Enhanced chemiluminescence (Pierce, MA, USA) was used to visualize the bands.

### Cell culture and treatment

The H9C2 cell line was kindly provided by Dr. Xiaoqiu Tan (Cardiovascular Research Institute, Southwest Medical University, Luzhou, China). H9C2 cells were cultured according to the usual protocol reported previously (14). Additionally, pretreatment of cells with 10 μM NG was performed for 2 h before exposure to 33 mM glucose (HG) for 24 h.

### ROS detection

Tissue sections or H9C2 cells were incubated for 30 min at 37°C using dihydroethidium (DHE; KeyGEN Biotech, Nanjing, China) or 2,7-Dichlorodi -hydrofluorescein diacetate (DCFH-DA; Beyotime Biotechnology), respectively. Following three washes with PBS, images of the tissue sections and cells were obtained using a fluorescence microscope. Graphical analysis software was applied to determine the mean fluorescence intensity.

### Flow cytometric analysis

Apoptosis was analyzed by flow cytometry (15). Cells were trypsinized, harvested, washed twice with cold PBS, and centrifuged. The supernatant was removed, and the cells were resuspended in 1 ml of binding buffer. Cells were vortexed gently, incubated for 10 min at room temperature in the dark, and stained using 5 μl Annexin V-FITC. Cell staining was performed with a 5 μl propidium iodide (PI) solution for 5 min at room temperature in the dark. Cells were resuspended in 500 μl PBS and gently vortexed. Cell analysis was carried out by flow cytometry within 1 h.

### Statistics

Measurements are expressed as means ± standard deviations. Analysis of variance was applied to determine the statistical significance between groups. Individual points were analyzed for statistical significance using Student's *t*-test. Statistics were conducted using SPSS 17. A value of *P* < 0.05 was considered statistically significant.

## Results

### NG ameliorated hyperglycemia and pathologic damage to cardiac tissue in diabetic mice

**Figure 1:**
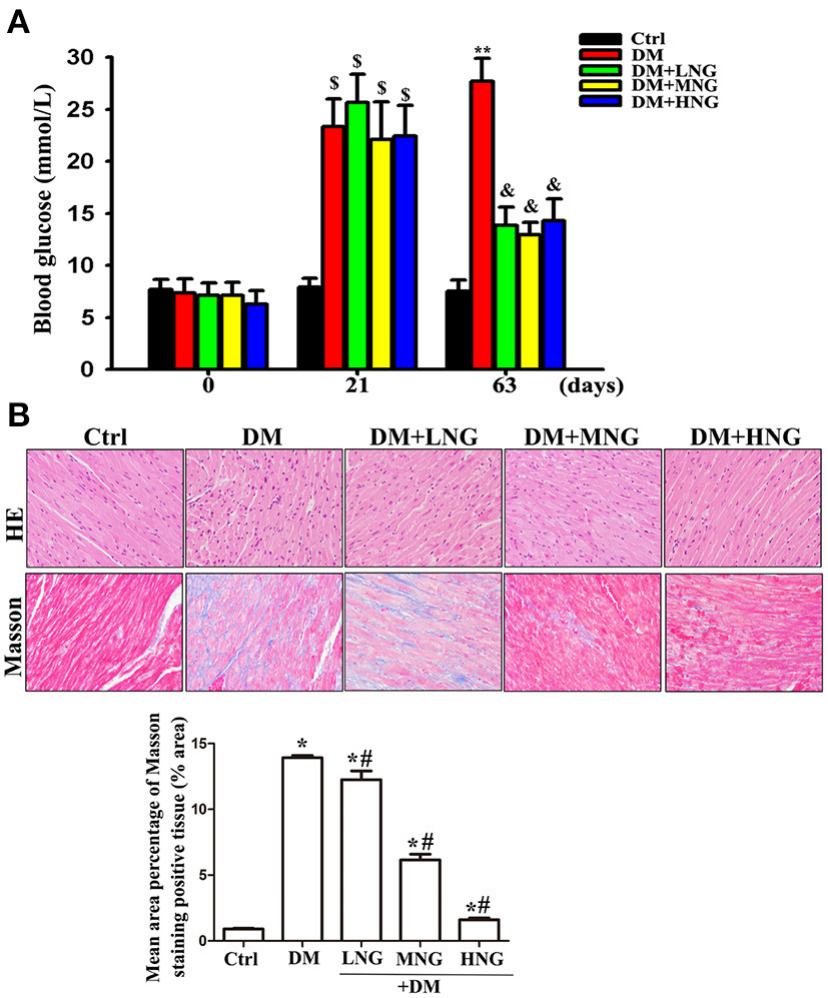
NG improves hyperglycemia and pathologic damage to cardiac tissue in diabetic mice. **(A)** Blood glucose of mice from different treatments. Data are presented as the mean ± SD (*n* = 6). ^$^*P* < 0.05 vs. corresponding groups in 0 day; **P < 0.05 vs. Ctrl group in 63 days, ^&^*P* < 0.05 vs. DM group in 63 days. **(B)** Representative images of HE or Masson staining for each group, light microscope (400 ×). The data are presented as mean ± SD (*n* = 3). *P < 0.05 vs. Ctrl group; ^#^*P* < 0.05 vs. DM group.

**Figure 2:**
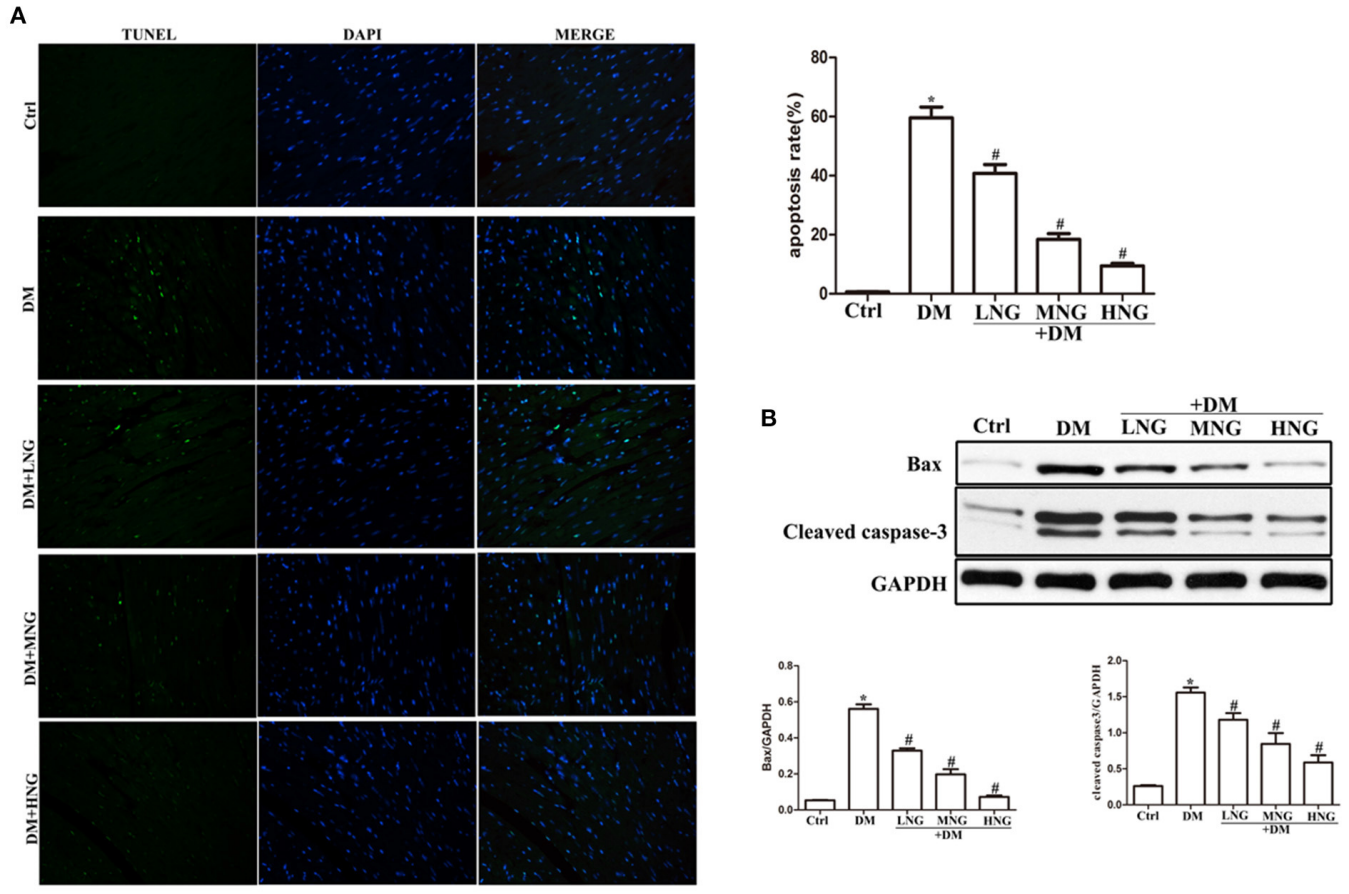
NG reduces apoptosis of cardiomyocytes in cardiac tissues of diabetic mice. **(A)** Representative images of TUNEL staining for each group, fluorescence microscope (200×). Data are presented as the mean ± SD (*n* = 3). *P < 0.05 vs. Ctrl group; ^#^*P* < 0.05 vs. DM group. **(B)** Quantitative analysis of the protein expression of Bax and cleaved caspase-3 in cardiac tissues. Data are expressed as mean ± SD (*n* = 3), *P < 0.05 vs. Ctrl group; ^#^*P* < 0.05 vs. DM group.

The blood glucose level of the STZ-treated groups at 21 days significantly increased compared to day 0 (*P* < 0.05, Figure 1A). After 63 days, blood glucose in the DM group was also significantly elevated compared to the Ctrl group (*P* < 0.05). In contrast, the differences in blood glucose after NG interventions were significantly reduced compared with the DM group (*P* < 0.05). HE and Masson staining demonstrated that cardiac structures were ordered in the Ctrl group, with a clear outline of tissues and collagen fibers in the interstitium (Figure 2B). On the contrary, degeneration, necrosis of some cardiomyocytes, disordered arrangement of cells, and most of the collagen fibers in the interstitium were observed in the DM group. These morphological injuries were improved in the DM groups treated with the different doses of NG (Figure 2B).

### NG reduced cardiomyocyte apoptosis in the cardiac tissues of diabetic mice

TUNEL staining was used to detect cardiomyocyte apoptosis. The apoptosis rate in the DM group was significantly increased compared with the Ctrl group (*P* < 0.05, Figure 2A). Treatment with the different doses of NG significantly reduced the apoptosis rate (*P* < 0.05). Cardiomyocyte apoptosis is a significant factor inducing cardiac dysfunction (16). Therefore, apoptosis-related signal proteins were evaluated. Induction of hyperglycemia prominently increased Bax and cleaved caspase-3 compared with the Ctrl group (*P* < 0.05, Figure 2B). By contrast, the expressions of Bax and cleaved caspase-3 proteins were inhibited by treatment with the different NG doses (*P* < 0.05).

### NG inhibited the expression of inflammatory cytokines in the cardiac tissues of mice with diabetes

**Figure 3:**
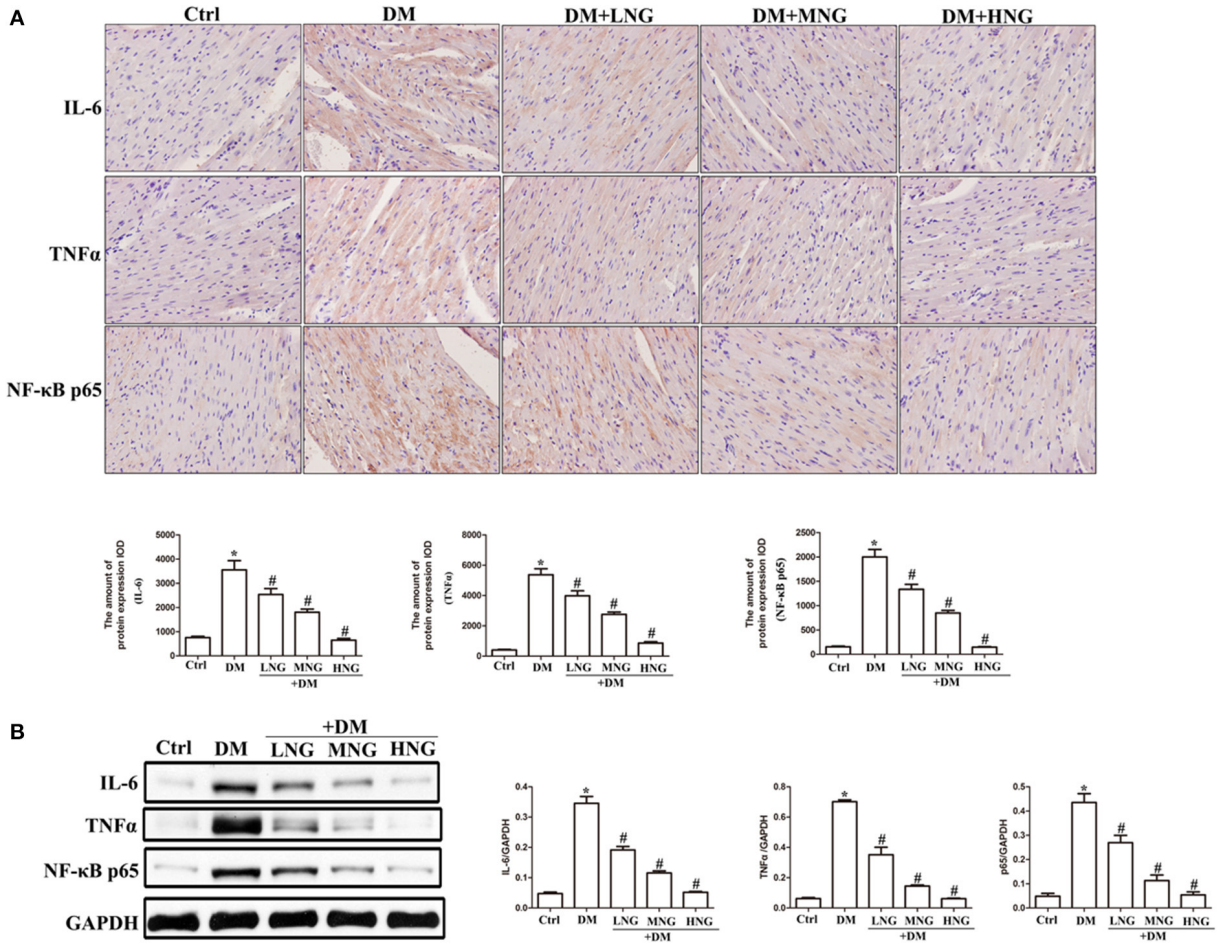
NG inhibites the expression of inflammatory cytokines in cardiac tissues of diabetic mice. **(A)** Representative immunohistochemical staining of IL-6, TNF-α, and NF-κB p65 in cardiac tissues. Data are expressed as mean ± SD (*n* = 3), *P < 0.05 vs. Ctrl group; ^#^*P* < 0.05 vs. DM group. **(B)** Quantitative analysis of the protein expression of IL-6, TNF-α, and NF-κB p65 in cardiac tissues. Data are expressed as mean ± SD (*n* = 3), *P < 0.05 vs. Ctrl group; ^#^*P* < 0.05 vs. DM group.

The images of cardiac sections from the groups stained for IL-6, TNF-α, or NF-κB p65 are shown in Figure 3A. As demonstrated by the quantitative analysis results, apparent upregulation of IL-6, TNF-α, and NF-κB p65 was observed in the DM group compared with the Ctrl group (*P* < 0.05, Figure 3A). Additionally, NG at the different doses used to treat diabetic mice reduced the expression of IL-6, TNF-α, and NF-κB p65 in a dose-dependent manner (Figure 3A), as well as decreased the IL-6, TNF-α, and NF-κB p65 protein levels (Figure 3B).

### NG reduced diabetes-induced oxidative stress in cardiac tissues of mice with diabetes

**Figure 4:**
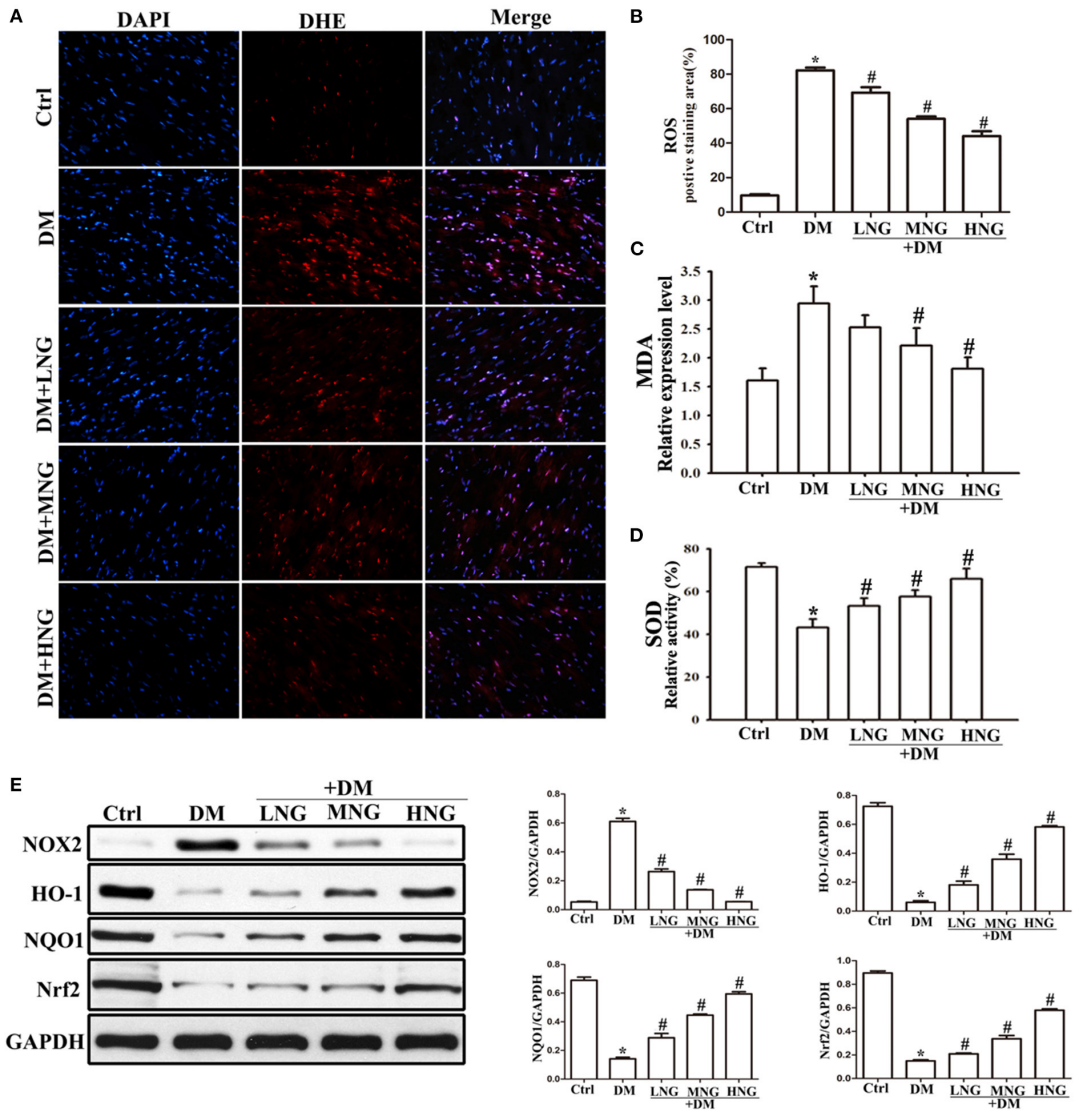
NG reduces diabetes-induced oxidative stress in cardiac tissues of diabetic mice. **(A)** Representative images of DHE staining for each group, fluorescence microscope (200×). **(B)** Quantitative analysis of the number of DHE fluorescence cells in cardiac tissue samples. Data are expressed as mean ± SD (*n* = 3), *P < 0.05 vs. Ctrl group; ^#^*P* < 0.05 vs. DM group. **(C)** The level of malondialdehyde (MDA) from different treatments in cardiac tissue. Data are expressed as mean ± SD (*n* = 3), *P < 0.05 vs. Ctrl group; ^#^*P* < 0.05 vs. DM group. **(D)** The level of superoxide dismutase (SOD) of different treatments in cardiac tissue. Data are expressed as mean ± SD (*n* = 3), *P < 0.05 vs. Ctrl group; ^#^*P* < 0.05 vs. DM group. **(E)** Quantitative analysis of the protein expression of NOX2, HO-1, NQO1, and Nrf2 in cardiac tissues. Data are expressed as mean ± SD (*n* = 3), *P < 0.05 vs. Ctrl group; ^#^*P* < 0.05 vs. DM group.

The images of the cardiac sections stained with DHE from the different groups are shown in Figure 4A. Quantitative analysis demonstrated a significant ROS upregulation in the DM group compared with the Ctrl group (*P* < 0.05, Figure 4B). In contrast, the ROS levels in the different NG groups were reduced dose-dependent compared to the DM group (*P* < 0.05, Figure 4B). Furthermore, NG treatment resulted in decreased MDA levels (a biomarker of oxidative damage) in a dose-dependent manner (Figure 4C), together with increased antioxidant SOD enzyme activity (Figure 4D). Nrf2 was previously shown to have a significant effect in inducing phase II detoxifying enzymes, for example, HO-1 and NQO1 (17). Therefore, Figure 4E indicates that Nrf2 and its downstream signaling proteins HO-1 and NQO1 are down-regulated in the DM group compared with the Ctrl group (*P* < 0.05), whereas they are elevated by NG treatment. Since NOX2 is the primary source of ROS, these results showed that NOX2 protein expression was up-regulated in the DM group compared with the Ctrl group (*P* < 0.05, Figure 4E) but was reduced by NG treatment.

### NG reduced HG-induced oxidative pressure and apoptosis in H9C2 cells

**Figure 5:**
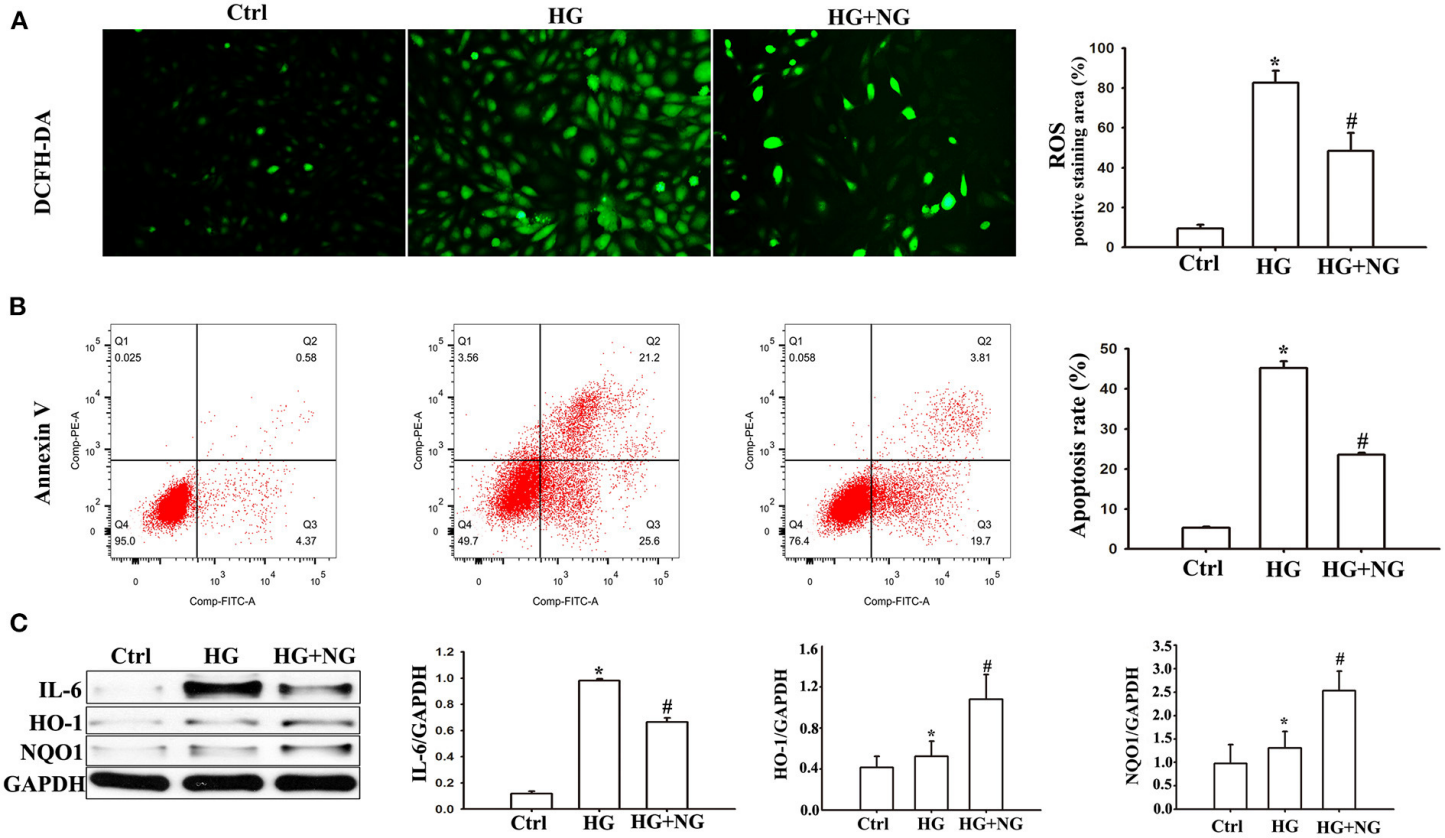
NG reduced HG-induced oxidative stress and apoptosis in H9C2 cells. **(A)** Representative images of DCFH-DA staining for each group, fluorescence microscope (200×). Data are expressed as mean ± SD (*n* = 3), *P < 0.05 vs. Ctrl group; ^#^*P* < 0.05 vs. DM group. **(B)** Evaluation of apoptosis by flow cytometry using Annexin V staining. Data are expressed as mean ± SD (*n* = 3), *P < 0.05 vs. Ctrl group; ^#^*P* < 0.05 vs. DM group. **(C)** Quantitative analysis of the protein expression of IL-6, HO-1, and NQO1 in H9C2 cells. Data are expressed as mean ± SD (*n* = 3), *P < 0.05 vs. Ctrl group; ^#^*P* < 0.05 vs. DM group.

Reactive oxygen species analysis by DCFH-DA staining demonstrated a much higher percentage of ROS in H9C2 cells cultured under HG conditions compared to the Ctrl group (*P* < 0.05, Figure 5A). However, there was a significantly decreased percentage of ROS cultured in HG after NG treatment (*P* < 0.05, Figure 5A). The apoptosis analyses by flow cytometry showed a considerable increase in the percentage of apoptotic cells among the H9C2 cells cultured under HG conditions compared to the Ctrl group (*P* < 0.05, Figure 5B). For comparison, the percentage of apoptotic cells cultured in HG was markedly reduced after treatment with NG (*P* < 0.05, Figure 5B). In addition, significant changes in pro-inflammatory cytokine and antioxidant proteins were also detected. IL-6 expression increased in the DM group compared with the Ctrl group (*P* < 0.05, Figure 5C), while NG treatment reduced IL-6 expression compared to the DM group (*P* < 0.05, Figure 5C). The expression of HO-1 and NQO1 increased in the DM group compared with the Ctrl group (*P* < 0.05, Figure 5C), while the NG treatment reduced the expression of HO-1 and NQO1 compared to the DM group (*P* < 0.05, Figure 5C). These results indicated that the protective effects of NG were probably related to its antioxidant and anti-inflammatory characteristics.

### NG regulated NF-κB and Nrf2 expression in H9C2 cells under HG condition

**Figure 6:**
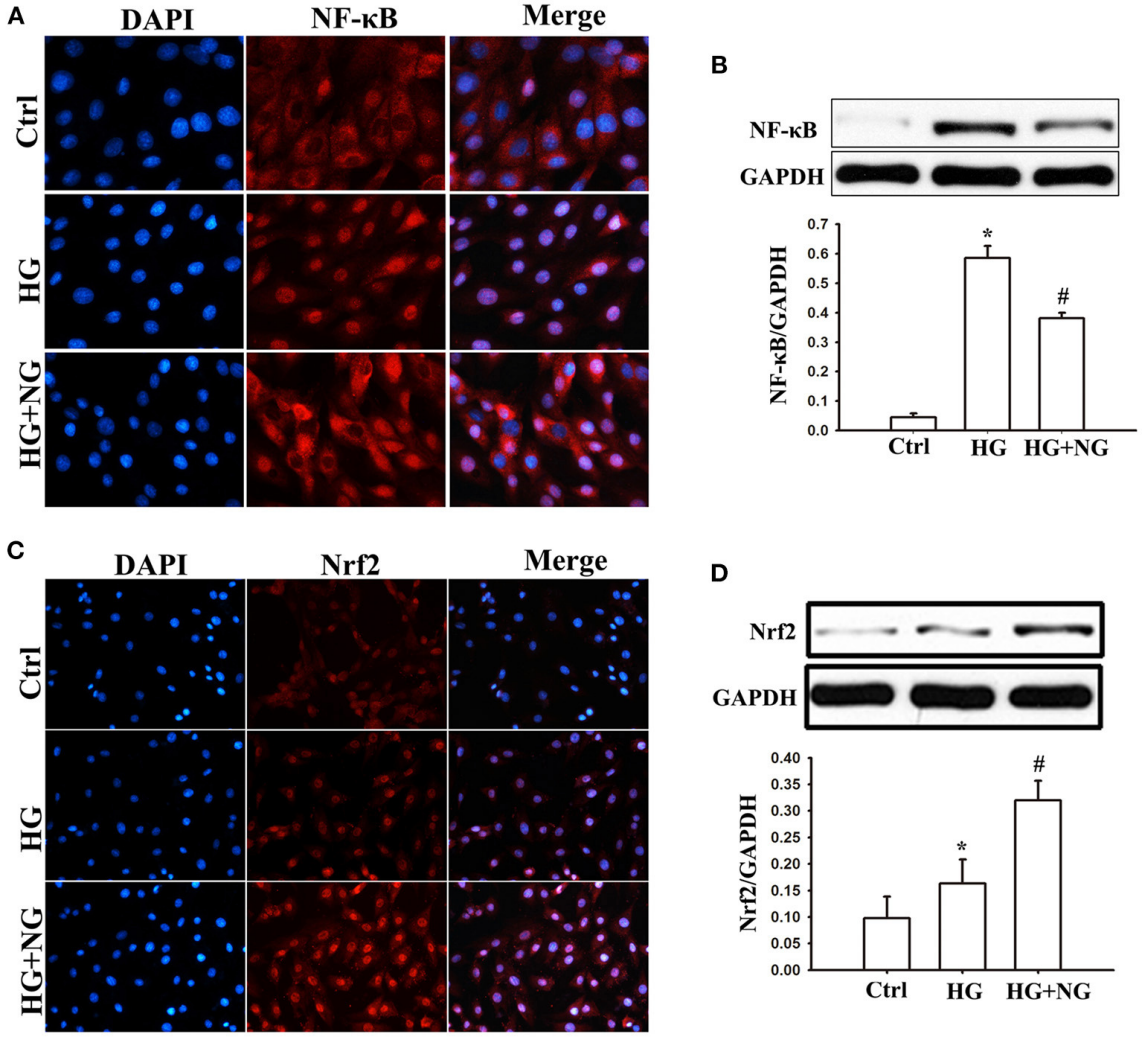
NG regulates the expression of NF-κB and Nrf2 in H9C2 cells under the HG condition. **(A)** Representative immunofluorescence staining of NF-κB for each group, fluorescence microscope (200×). **(B)** Quantitative analysis of the NF-κB protein expression in H9C2 cells. Data are expressed as mean ± SD (*n* = 3), *P < 0.05 vs. Ctrl group; ^#^*P* < 0.05 vs. DM group. **(C)** Representative immunofluorescence staining of Nrf2 for each group, fluorescence microscope (200×). **(D)** Quantitative analysis of the Nrf2protein expression in H9C2 cells. Data are expressed as mean ± SD (*n* = 3), *P < 0.05 vs. Ctrl group; ^#^*P* < 0.05 vs. DM group.

The influence of NG on NF-κB and Nrf2 activation was analyzed to determine the signaling pathways responsible for inflammatory cytokines and oxidative stress. Analysis of immunostaining data indicated that more positive staining for NF-κB p65 was observed in the nucleus under the HG condition compared to the Ctrl group (Figure 6A). At the same time, NG treatment reduced the positive staining for NF-κB p65 compared to the untreated HG group (Figure 6A). Consistent with this, NF-κB p65 protein expression in the nucleus was significantly suppressed by NG under the HG condition (*P* < 0.05, Figure 6B). Furthermore, analysis of the immunostaining showed less positive staining for Nrf2 in the nucleus under the HG conditions compared to the Ctrl group (Figure 6C). At the same time, NG treatment increased the positive staining for Nrf2 compared with the untreated HG group (Figure 6C). NG significantly upregulated the Nrf2 protein expression in the nucleus under the HG condition (*P* < 0.05, Figure 6D).

## Discussion

Naringenin has been reported to have many cardioprotective activities (7, 18, 19). Although NG has been reported to improve high glucose-induced hypertrophy of cardiomyocytes in diabetic models (20, 21), the mechanism by which it exerts these protective effects against diabetic cardiomyopathy is not fully understood. Progressive oxidative stress and inflammation development are prominent markers of diabetic cardiomyopathy (22, 23). Therefore, inhibiting oxidative stress and inflammation during disease progression may be a promising strategy for treating diabetic cardiomyopathy.

**Figure 7:**
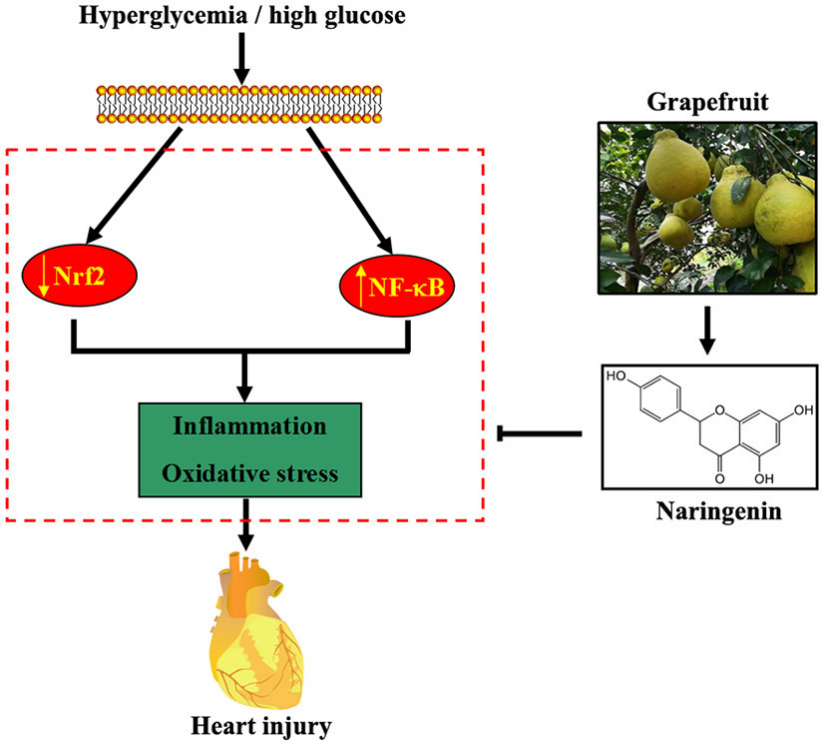
Proposed pathway for the cardioprotective effect of NG against diabetes-induced myocardial injury. Hyperglycemia or HG reduces Nrf2 expression and induces NF-κB expression, resulting in oxidative stress and inflammation. NG, a flavonoid abundant in grapefruit, partially attenuates diabetes-induced heart injury by activating the Nrf2 pathway and inhibiting the NF-κB pathway.

Several lines of evidence have shown that NG exerts antioxidant and anti-inflammatory pharmacological activities (24, 25). Our previous study shows that NG ameliorates fibrosis by down-regulating Rho A/Rho-associated protein kinase (ROCK) signaling pathways in diabetic nephropathy mice (26). Furthermore, NG induced the expression of the antioxidant protein HO-1, reducing endothelial cell apoptosis under the HG condition (15). In the current study, NG improved diabetes-induced myocardial injury by reducing oxidative stress and inflammation by regulating the Nrf2 and NF-κB signaling pathways (Figure 7).

Chronic persistent inflammation can be one of the main reasons that hyperglycemia leads to changes in myocardial structure and function (16, 27). NF-κB is a vital transcription regulator for proinflammatory cytokine genes, including IL-6 and TNF-α (28). The hearts of diabetic rats were characterized by increased NF-κB p65, IL-6, and TNF-α expression (29). Sophocarpine inhibition of NF-κB-mediated inflammation attenuated diabetic cardiomyopathy (30). In the present study, NG reduced the hyperglycemia-induced expression of proinflammatory cytokines and NF-κB. Therefore, the mediation of the protective effect of NG was based on inhibiting the NF-κB pathway.

In addition to cardiac inflammation, hyperglycemia-induced oxidative stress also affects the progression of diabetic cardiomyopathy. Enhancing antioxidant defense may protect against diabetes-induced cardiac dysfunction (31). As one of the most important transcription factors, Nrf2 exerts antioxidant and anti-apoptotic effects by activating multiple antioxidant genes, including HO-1 and NQO1 (6, 32). Our study showed that hyperglycemia increased ROS and MDA levels *in vivo*. Furthermore, NG treatment increased antioxidant enzymes (HO-1, NQO1, and SOD), reduced ROS generation, and activated Nrf2 in cardiac tissues from diabetic mice. Therefore, the results suggested that the Nrf2 pathway might be involved in the cardioprotective effect of NG. In particular, exposure to HG caused slightly higher Nrf2 expression than the control group. The findings differ from our *in vivo* study in which hyperglycemia suppressed Nrf2 expression. The cause of this discrepancy may be attributed to the duration of stimulation. We hypothesize that a short exposure of the cell to HG induces the compensatory protection mechanism. Therefore, Nrf2 is activated to exert its antioxidant stress effect. However, when animals are exposed to longer-term hyperglycemia, the antioxidant system will be weakened. Thus, Nrf2 expression is down-regulated by long-term hyperglycemia.

Oxidative stress and inflammation lead to cardiomyocyte apoptosis (33, 34). Therefore, the effect of NG on cardiomyocyte apoptosis was observed both *in vitro* and *in vivo*. In diabetic mice, NG treatment reduced hyperglycemia-induced cell apoptosis. Subsequently, NG also downregulated the expression of cleaved caspase-3 and the pro-apoptotic protein Bax. These data showed that inhibition of cardiomyocyte apoptosis could be one of the critical mechanisms of NG to improve diabetes-induced cardiac dysfunction. Furthermore, subsequent cardiac interstitial collagen deposition was attenuated by NG treatment.

This study found that NG ameliorated myocardial injury in diabetic mice. NG inhibited pro-inflammatory cytokines, ROS level, and NF-κB expression, while antioxidant enzymes and Nrf2 expression were significantly enhanced. The main limitation of this study was the lack of application of signal pathway inhibitors or gene knockdown in *in vitro* experiments. There is no direct evidence that NG inhibits oxidative stress, inflammation, or apoptosis by regulating Nrf2 or NF-κB. However, in support of our findings, inhibition of Nrf2 has been reported to suppress the NG-induced protective effect induced by NG in cardiac fibroblasts and vascular endothelial cells (15, 35). We will confirm these observations in future studies.

This study demonstrated that NG ameliorated myocardial injury in STZ-induced diabetic mice by improving Nrf2-mediated antioxidant stress and reducing NF-κB-mediated inflammation. Furthermore, targeting Nrf2 and NF-κB may be an important therapeutic strategy for reducing diabetic complications.

## Data availability statement

The original contributions presented in the study are included in the article/supplementary material, further inquiries can be directed to the corresponding author.

## Ethics statement

The study was approved by the Ethical Committee of Chongqing University Cancer Hospital.

## Author contributions

YH and SW performed the experiment and wrote the paper. HS wrote the paper. YL analyzed the data. JF reviewed and revised the paper. All authors contributed to the article and approved the submitted version.

## Funding

This research was funded by Luzhou Municipal People's Government - Southwest Medical University Science and Technology Strategic Cooperation (2021LZXNYD-J33), the Technology Innovation and Application Development Program of the Chongqing Municipal Science and Technology Bureau (cstc2018jscx-msybX0157), and the Chongqing Kewei Joint Medical Research Project (2021MSXM337).

## Conflict of interest

The authors declare that the research was conducted in the absence of any commercial or financial relationships that could be construed as a potential conflict of interest.

## Publisher's note

All claims expressed in this article are solely those of the authors and do not necessarily represent those of their affiliated organizations, or those of the publisher, the editors and the reviewers. Any product that may be evaluated in this article, or claim that may be made by its manufacturer, is not guaranteed or endorsed by the publisher.
